# Are cannabis-using and non-using patients different groups? Towards understanding the neurobiology of cannabis use in psychotic disorders

**DOI:** 10.1177/0269881118760662

**Published:** 2018-03-29

**Authors:** Musa Basseer Sami, Sagnik Bhattacharyya

**Affiliations:** 1 Institute of Psychiatry, Psychology & Neuroscience, King’s College London, UK; 2 Lambeth Early Onset Inpatient Unit, Lambeth Hospital, South London and Maudsley NHS Foundation Trust, UK

**Keywords:** Cannabis, psychosis, schizophrenia, MRI, endocannabinoid

## Abstract

A substantial body of credible evidence has accumulated that suggest that cannabis use is an important potentially preventable risk factor for the development of psychotic illness and its worse prognosis following the onset of psychosis. Here we summarize the relevant evidence to argue that the time has come to investigate the neurobiological effects of cannabis in patients with psychotic disorders. In the first section we summarize evidence from longitudinal studies that controlled for a range of potential confounders of the association of cannabis use with increased risk of developing psychotic disorders, increased risk of hospitalization, frequent and longer hospital stays, and failure of treatment with medications for psychosis in those with established illness. Although some evidence has emerged that cannabis-using and non-using patients with psychotic disorders may have distinct patterns of neurocognitive and neurodevelopmental impairments, the biological underpinnings of the effects of cannabis remain to be fully elucidated. In the second and third sections we undertake a systematic review of 70 studies, including over 3000 patients with psychotic disorders or at increased risk of psychotic disorder, in order to delineate potential neurobiological and neurochemical mechanisms that may underlie the effects of cannabis in psychotic disorders and suggest avenues for future research.

## Introduction

Psychotic illnesses have an annual prevalence of around 0.4% of the population and a cost to the economy of £13.5 billion a year in England alone (Kirkbride et al., 2012). Early-onset psychosis may herald chronic illness, such as schizophrenia or schizoaffective disorder, with considerable impact on the individual’s functioning, social and occupational opportunities. Consequently, better understanding of early psychosis is critical to the development of preventative and treatment strategies. Comorbid cannabis use is a particularly important problem for the clinician treating patients with psychosis, as it is present in around 30–40% of patients with early psychosis, constituting a considerable proportion of their caseload (Myles et al., 2015), and is associated with poorer prognosis (Patel et al., 2016; Schoeler et al., 2016a, b, c, d).

This article will restrict itself to the association between cannabis and psychotic disorders. When discussing the association between cannabis and psychosis a clear distinction must be made between several psychosis outcomes: (a) risk of developing enduring psychotic disorder, (b) risk of relapse once a psychotic disorder has developed and (c) the transient psychotomimetic states that cannabis induces (Murray et al., 2017). In particular, transient psychotomimetic states are common, self-limiting, non-clinical entities, experienced in around 15% of users (Thomas, 1996); demonstrable in healthy individuals when administered phytocannabinoids (D’Souza et al., 2004), with increased propensity with psychotic disorders or risk of psychosis (Henquet et al., 2006, 2010; Vadhan et al., 2017; Verdoux et al., 2003), they are not the focus of this article, wherein we focus on the relationship between cannabis and outcomes related to the development of psychotic disorder and its relapse. In the first section we present a critical review as an overview of the epidemiological association between cannabis and psychotic disorder, and go on to discuss differences between patients with established psychotic disorder who use cannabis and those who do not. In the second section, in order to advance understanding of the neurobiological association of cannabis and psychotic disorder, we undertake a systematic search to delineate whether neurobiological differences exist between groups.

### I. Epidemiological understandings to date relating to psychotic disorder

It is worth bearing in mind, in the discussion that follows, that cannabis is not a homogenous entity and is constituted of a variety of chemical constituents which may have varying and, at times, opposing effects (Bhattacharyya et al., 2012a; Colizzi and Bhattacharyya, 2017). Particular interest has focused on two plant cannabinoids: (a) Δ-9-tetrahydrocannabinol (THC) which has anxiolytic effects at lower doses, whereas at higher doses typically demonstrates anxiogenic and psychotomimetic effects (Bhattacharyya et al., 2010, 2012b, 2017), and (b) cannabidiol (CBD), which typically demonstrates anxiolytic properties (Crippa et al., 2011). Yet there are over 100 cannabinoid constituents identified, and the postulated role of the minor phytocannabinoids in modulating these effects (‘the entourage effect’) remains to be fully elucidated (Mechoulam et al., 2014; Russo, 2011). This points to a limitation in the epidemiological evidence where potency of cannabinoid constituents is infrequently considered, with marked variation noted between sites (Potter et al., 2008; Vergara et al., 2017) and marked variation between actual and labelled cannabinoid content (Bonn-Miller et al., 2017; Vandrey et al., 2015). A further significant limitation in the epidemiological evidence is in the varying definition for ‘cannabis user’ – for example, whether users are defined by lifetime or current use; and ‘heavy use’ – for example whether this relates to frequency or duration of use (Marconi et al., 2016). Furthermore, whereas laboratory-based studies have established alternative pharmacodynamic profiles between methods of cannabinoid ingestion (inhaled, intravenous, oral) (Huestis, 2007; Radhakrishnan et al., 2014; Sherif et al., 2016), this has not been yet considered in relation to the risk of psychotic disorder or its relapse. Hence no firm conclusions can be drawn about this.

#### Cannabis and psychosis risk

Much of the focus to date has been towards examining whether cannabis use is a risk factor for psychotic illness (Ksir and Hart, 2016). This has historically been complicated by the ‘self-medication hypothesis’. This posits that individuals with psychosis use cannabis to alleviate symptoms (reverse causality), hence that psychosis predicts cannabis use and explains a proportion of the association (Ferdinand et al., 2005). However, evidence of a temporal relationship, with cannabis use preceding the onset of psychosis, has been credibly demonstrated in a number of studies (Moore et al., 2007), which argues against the reverse causality hypothesis. For example, in a re-analysis of the Swedish conscript study, an increase in psychotic incidence in historical cannabis users continued to be reported over 5 years after the initial assessment of cannabis use (Zammit et al., 2002). Similar results of cannabis use preceding increases in psychosis risk have been shown in New Zealand, Dutch and British cohorts (Arseneault et al., 2002; Gage et al., 2015; Van Os et al., 2002). Structural equation modelling has further demonstrated (Fergusson et al., 2005) that the association between cannabis use and psychosis reflects the effect of cannabis use on psychotic symptoms rather than the other way round, although another study that employed a sibling-pair design (McGrath et al., 2010) suggested that the relationship may be more nuanced. Evidence from McGrath et al. suggested that those who are vulnerable to developing psychosis might also be more likely to start using cannabis, which in turn may increase the likelihood of their developing a psychotic disorder subsequently.

Longitudinal studies have also demonstrated a dose-response relationship between cannabis use and psychosis risk: Moore and colleagues undertook a major meta-analysis which demonstrated an increased Odds Ratio (OR) of 1.41 for cannabis users (OR 2.09 in more frequent users) of developing a psychotic outcome (Moore et al., 2007). It has been noted that the overall OR is small (Haney and Evins, 2016), although a more recent meta-analysis also demonstrated an increased OR of 3.90 for most severe users compared with non-users (Marconi et al., 2016). It has been argued that the dose-related association between cannabis use and psychosis risk may not be related to a causal association, but may represent an attempt at self-medication of an impaired neurodevelopmental profile in the years preceding illness (Haney and Evins, 2016). To our knowledge, in the absence of reported data from large longitudinal cohorts, this has yet to be tested directly. However, the literature appears to suggest that neurodevelopmental liability appears to be a less common feature in patients who develop psychosis and use cannabis (discussed below).

A further important consideration is a shared vulnerability for psychotic disorders and use of cannabis through genetic vulnerability or stress (Haney and Evins, 2016; Ksir and Hart, 2016). An important methodological limitation of observational studies that precludes against definitive conclusions being drawn is inadequate adjustment for the plethora of potential confounders: around 60 different confounders have been identified, and Moore and colleagues noted that residual confounders can never be completely eliminated (Moore et al., 2007). A more recent review noted that an increased risk of psychosis in cannabis users remains when extensive confounders are adjusted for (such as age, sex, substance misuse, tobacco use), although certain confounders such as childhood trauma and genetic variability have been more infrequently looked at (Gage et al., 2016). Large-scale studies demonstrate higher polygenic risk of schizophrenia to be associated with cannabis, supporting shared genetic vulnerability, but this accounts for a small fraction of the variance of cannabis use overall (0.47–0.49%) (Power et al., 2014; Reginsson et al., 2017). Evidence from studies that have used statistical modelling (Fergusson et al., 2005) and sibling-pair design (McGrath et al., 2010) suggests that the association between cannabis use and psychosis is unlikely to be explained by residual confounding factors such as shared vulnerability (e.g. genetic) (Schoeler et al., 2016b) that may not have been measured in longitudinal studies.

It has been therefore argued that cannabis represents a component cause for enduring psychotic disorder, that is, by itself neither necessary nor sufficient to cause disorder but implicated in the patho-aetiology of illness alongside other factors which increase psychosis risk (Arseneault et al., 2004; Gage et al., 2016). One approach is to consider whether transition to psychosis occurs in enriched samples, that is, those at higher risk of developing disorder in cannabis users as opposed to non-users. This approach is made complicated by the fact that at least one study has shown that those who self-present at clinical high risk are likely to stop using cannabis due to the distressing nature of the symptoms (Valmaggia et al., 2014). Although earlier studies had not shown an association between cannabis use and psychosis transition (Auther et al., 2012; Corcoran et al., 2008), more recent larger studies considering abuse or cannabis-induced attenuated psychotic symptoms have shown an association between cannabis and psychosis transition (Auther et al., 2015; McHugh et al., 2017), although in Auther et al. this only remains at the trend level (*p*=0.064) after adjusting for alcohol use.

Finally, a further argument against a causal association between cannabis and psychosis relates to the incidence of psychosis (Hill, 2015). It has been demonstrated that, despite an increase in potency of cannabis use over several decades, this has not led to an expected increase in the incidence of psychotic disorders (Frisher et al., 2009; Kirkbride et al., 2012), thus rendering a causal association unlikely. This remains a contentious point – some authors have pointed out that despite increasing rates of obesity, a well-established risk factor for cardiovascular health, cardiovascular health outcomes have improved in recent times in the Western world (Large et al., 2015). Thus the epidemiological relationship between risk factor and outcome does not hold if there is an intervening third factor (e.g. improved availability of cardiovascular treatments). Recently some data have emerged suggesting that psychotomimetic experiences in cannabis users, which may be a precursor to psychosis risk, lead to discontinuation of cannabis use, which may in turn offset risk of transition to psychosis in high-risk groups (Sami et al., 2018; Valmaggia et al., 2014; van Gastel et al., 2014).

#### Cannabis use and psychotic relapse

In addition to increasing the risk of onset of psychosis, cannabis use also has an adverse effect on outcome in those who develop psychosis. Of the 50089 participants of the Swedish conscript study, 401 (0.8%) had schizophrenia-related inpatient admissions in the subsequent 34 years. Of those in whom baseline cannabis data were available, 78/357 (20%) had a history of cannabis use. This group had had more hospital admissions compared with never users over the 34-year period (10 vs. four) and had spent a year longer in hospital compared with the never users (547 days vs. 184) (Manrique-Garcia et al., 2014).

Other studies confirm adverse prognostic features of cannabis use. In patients with an established psychotic illness, continued cannabis use has been shown to be associated with adverse outcomes. A recent meta-analysis using pooled data from over 16500 patients with psychosis, who were at various stages of psychotic illness, demonstrated that continued cannabis use is associated with greater risk of relapse, hospitalization and longer inpatient admissions (Schoeler et al., 2016a). Discontinuing cannabis use appeared to decrease the risk of relapse to a level that was similar to patients who had never used cannabis. That cannabis use at presentation with first-episode psychosis (FEP) is associated with increased risk of relapse subsequently was further independently confirmed by 5-year follow-up of over 2000 FEP patients in South London (Patel et al., 2016). This study suggested that those with cannabis use at onset of FEP spent on average 35 additional days in hospital over 5 years compared with those without history of cannabis use. However, not all patients who develop psychosis following cannabis use continue using the drug following onset of their illness. This was investigated in a separate study, which demonstrated that outcomes are worse in those reporting frequent and continued use of high-potency cannabis (Schoeler et al., 2016a) compared with those who had stopped, also suggesting a dose-response relationship.

A further prospective study across five UK sites (*n*=1027) following FEP patients for a year after referral to Early Intervention Services also demonstrated poorer outcome in cannabis-using patients which remained after adjusting for other substance misuse (Seddon et al., 2015). However, the authors did note that they could not completely exclude the possibility that increases in symptomatology led to increases in use. Furthermore, all of these studies also did not adjust for treatment compliance. This is particularly important, as a recent meta-analysis has demonstrated that history of cannabis use may be associated with 150% increase in the risk of treatment non-adherence (Foglia et al., 2017), consistent with independent evidence (Schoeler et al., 2017a) as well as evidence that impaired treatment adherence may mediate the association between cannabis use and poor outcome in those with FEP (Schoeler et al., 2017b).

Similar to the association between cannabis use and psychotic onset, the association between cannabis use and psychotic relapse must adjust for a plethora of potential confounders, including tobacco smoking, other substance misuse, medication non-adherence, personality and genetic traits which may aggregate in the cannabis-using group. The difficulty in ensuring this across all studies relates to the limitations of observational methods in disentangling known and unknown confounders. Evidently, due to ethical reasons, one cannot administer cannabis in double-blind randomized controlled trials to determine psychosis outcome. However, recent evidence employing a quasi-experimental design within a longitudinal design demonstrates that alternative explanations and residual confounders do not fully explain the relationship between cannabis and psychotic relapse, and suggest that increased risk of relapse is contributed to by the cannabis itself (Schoeler et al., 2016b, c, d).In particular, this study demonstrated that even after controlling for the effect of the most important factors that vary over the course of illness and may potentially affect outcome (such as adherence to medication for psychosis and use of other illicit drugs following onset of psychosis), as well as for premorbid confounding factors that remained stable over the course of illness (such as shared familial and genetic vulnerability, personality traits, history of childhood trauma, duration of untreated psychosis, expressed emotion and cannabis use history before the onset of psychosis), cannabis use remained a significant risk factor relapse. By comparing periods of cannabis use with periods of no use within the same individual, this study demonstrated that both change in cannabis use status (e.g. from being an user to a non-user or vice versa) and change in the pattern of cannabis use (e.g. from intermittent use to more regular cannabis use) adversely affects outcome in established psychosis, suggesting the existence of a biological gradient (dose-response relationship). Furthermore, the authors were able to rule out the possibility of reverse causality by demonstrating that cannabis use status and change in pattern of cannabis use predicted subsequent relapse and not vice versa.

There is some evidence for poorer treatment response in cannabis-using patients. In an experimental study, D2 receptor antagonists have been shown to inadequately prevent psychotic symptoms induced by administration of THC to stable patients (D’Souza et al., 2005). A systematic review suggests that conventional medications for psychosis (dopamine receptor antagonists) have some efficacy in psychotic patients with cannabis use, with some suggestion that clozapine, which is believed to have adjunctive non-dopaminergic action, is superior in treating psychotic symptoms and reducing cannabis cravings (Wilson and Bhattacharyya, 2015). However, this was demonstrated in studies with small sample sizes and did not directly compare treatment response in cannabis-using and non-using groups. One study of 161 previously drug-naïve non-affective FEP patients administered olanzapine, risperidone or haloperidol over 6 weeks demonstrated poor treatment response and disorganization in cannabis users (Pelayo-Terán et al., 2014). In a large naturalistic sample (*n*=2026) followed up to 5 years after presentation, cannabis use has been shown to be associated with an increased number of unique medications for psychosis prescribed, a proxy measure for decreased tolerability and poor treatment response in a naturalistic setting (Patel et al., 2016). However, this study did not adjust for poor treatment compliance and other substance misuse. Future studies in this area should therefore ensure adequate measures of compliance to ensure a precise measurement of the effect of cannabis use on response to treatment.

Consequently one arrives at the conclusion that, although limitations in the epidemiological evidence still exist, there is something in the biological substance of exogenously administered phytocannabinoids which potentiates psychotic disorders. This relationship appears to be more well established for those with established psychotic disorders (i.e. relapse) than psychosis risk, for which debates about the extent of a causal link remain. Hence, for patients with established psychotic disorders, it seems that the time has come to move beyond debates about associations of cannabis and psychosis to developing an understanding of the underlying neurobiology in order to develop interventions that work.

### Are cannabis-using and non-using patients different groups?

Why cannabis-using psychosis patients have a worse prognosis is as yet unclear. Emerging evidence suggests that cannabis-using psychosis patients may have distinct clinical, prognostic and neurocognitive features compared with non-users (Table 1). A large meta-analysis including over 22500 patients has shown that patients who develop psychosis following a history of cannabis use present almost 3 years earlier, whereas alcohol does not have the same effect (Large et al., 2011). A further meta-analysis revealed that adjusting for tobacco use did not change this finding (Myles et al., 2012).

**Table 1.** Differences between cannabis-using patients and non-using patients with early psychosis.

		Patients with early psychosis	
	*n*	Cannabis users	Non-cannabis users
Illness age of onset (Large et al., 2011)	22519	↓	↑
Risk of relapse (Schoeler et al., 2016a)	16500	↑	↓
Duration hospital stay (Schoeler et al., 2016a)	16500	↑	↓
Hospital admissions (Manrique-Garcia et al., 2014; Patel et al., 2016)	357 over 35 years; 2026 over 5 years	↑	↓
Medication used (Patel et al., 2016)	2026	↑	↓
Neurological soft signs (Bersani et al., 2002; Mallet et al., 2017; Mhalla et al., 2017; Ruiz-Veguilla et al., 2009);	264	↓	↑
Memory performance (Schoeler et al., 2015)	3261	↑	↓

Interestingly, poorer prognosis and younger age of onset in cannabis-using patients contrasts with the suggestion of their having a less impaired neurodevelopmental profile. Three lines of evidence converge to show this: (a) fewer neurological soft signs (NSS) in cannabis-using patients versus non-using patients (Bersani et al., 2002; Mallet et al., 2017; Mhalla et al., 2017; Ruiz-Veguilla et al., 2009); (b) improved neurocognitive performance in the cannabis-using group (Schoeler et al., 2015; Stirling et al., 2005); and (c) neuroimaging and other neurobiological measures such as increased hippocampal volumes in the cannabis-using group, which has been shown in some studies (Cunha et al., 2013; Koenders et al., 2015), although not shown in all studies (Malchow et al., 2013); discussed in further detail in the second section.

#### Neurological soft signs

Fewer NSS have been reported in FEP cohorts in heavy cannabis users. One study (*n*=92) demonstrated this in a group of daily cannabis users versus infrequent or non-existent use (Ruiz-Veguilla et al., 2009). Although the cannabis-using group had a preponderance of weekly cocaine users (32/51), there remained significantly fewer NSS when this was adjusted for. A more recent French study (*n*=61) demonstrated similar findings in daily or dependent cannabis users as opposed to less frequent users when tobacco, alcohol but not other substance misuse had been adjusted for (Mallet et al., 2017), whereas a Tunisian cohort (*n*=61) found findings in the same direction after adjusting for tobacco and alcohol (Mhalla et al., 2017). The difference between cannabis-using and non-using patients, with fewer NSS, does not appear to be limited to early psychosis and has been demonstrated in a cohort of chronic patients (Bersani et al., 2002). A further study of 112 patients, of whom 69 were followed up after 10 years, conversely did show increased NSS at presentation in cannabis-using patients, but showed neurocognitive sparing compared with non-users after 10 years (Stirling et al., 2005). This would argue against the suggestion of pre-existing differences and may suggest cannabis use has a protective role. However, given the evidence of increased neurocognitive impairment associated with cannabis use in non-clinical samples (see Lynskey and Hall, 2000; Meier et al., 2012; Schoeler et al., 2015), this may be unlikely in clinical samples and such an interpretation requires further replication and should be considered with caution. Given the paucity of longitudinal data, further studies are needed before definitive conclusions can be drawn.

#### Neurocognition

A meta-analysis of over 3200 patients with early psychosis showed better cognitive performance in cannabis-using patients in global memory, visual recall and recognition compared with those not using cannabis, with the authors suggesting that cannabis-using patients were a younger, less depressed and neurocognitively spared group (Schoeler et al., 2015). Other meta-analyses have similarly found improved neurocognitive profile in patients who use cannabis (Rabin et al., 2011; Yucel et al., 2012). One explanation for this could be the higher abilities required for acquisition and use of cannabis. In line with this it has been argued that improved executive function in cannabis-using patients would equate to improved social cognition. However, this has not been conclusively shown and preliminary findings have yielded contradictory results (Arnold et al., 2015; Helle et al., 2017).

Furthermore, the association between cannabis use and neurocognitive performance in the context of psychosis is not unidirectional, and recent studies have suggested a more nuanced view. One recent study of 268 FEP patients showed worse neurocognitive performance in cannabis-using patients, which was worse in those without a family history of psychosis compared with users with a family history (González-Pinto et al., 2016), whereas another major study (*n*=956) found worse outcomes in continued cannabis users, while lifetime users were neurocognitively spared (Meijer et al., 2012). While this may suggest an interaction between cannabis use and the propensity to develop psychotic disorder differentially affecting subgroups, further studies are required to disentangle the precise nature of this effect.

Taken together, for both NSS and neurocognition, the majority of evidence points towards decreased neurodevelopmental liability in cannabis users, although this is not unequivocal. Furthermore, this has been based upon mostly cross-sectional studies, hence causality cannot be established. These results require further replication in longitudinal studies to effectively disentangle the cannabis and psychosis interaction on neurological function.

Yet the phenotypic differences between cannabis-using patients and non-users suggest that cannabis-using patients may constitute a distinct group. This may be (i) due to the effect of cannabis itself; (ii) due to the effect of addiction or substance misuse on reward pathways; or (iii) this may represent cannabis-using patients having fewer pre-existing impairments and better cognitive abilities to start with. If cannabis users and non-users with psychosis are indeed distinct groups they may represent separate pathways to psychosis. Determining the biological underpinnings of such differences, particularly the neurochemical and neurophysiological abnormalities that may distinguish cannabis-using from non-using psychosis patients, may have implications in terms of unravelling potential alternative therapeutic targets in the cannabis-using group, a group with particularly worse outcomes.

### II. Unravelling neurobiological underpinnings between groups

In this and the following section we undertake a systematic review of neurobiological and neurochemical differences specifically between cannabis and non-using patients with psychosis as well as those at increased risk of developing psychosis. Previous reviews have extrapolated findings from preclinical studies and/or reported on findings from non-psychotic cannabis users (Colizzi et al., 2016; Murray et al., 2017; Radhakrishnan et al., 2014; Sami et al., 2015; van Winkel and Kuepper, 2014), or have not adequately differentiated cannabis from other substance misuse (Adan et al., 2017). In this section we focus on neurobiological measures (imaging and electrophysiological) in cannabis-using and non-using groups within the psychosis spectrum. In the third section we focus on neurochemical differences between groups.

#### Method

A systematic search strategy was undertaken with PubMed between November and December 2017 (last full search 28 December 2017), with results restricted to human studies. Search terms were: (cannabis OR marijuana) AND (psychosis OR schizo*) AND (MRI OR fMRI OR dopamine OR GABA OR glutam* OR CB1 OR CB1R OR endocannabinoid OR postmortem OR autopsy OR autoradiography OR EEG OR evoked potential OR event related potential OR ERP). Articles were selected by the following criteria: (a) human studies; (b) relevant to neurobiological measures of psychosis; (c) including a cannabis-using group of patients with psychotic disorder (P-C); (d) including a non-cannabis-using group of patients with psychotic disorder (P-NC). Some 325 articles were identified on initial search and abstracts reviewed. Identified articles were hand-searched for further articles and relevant references for reviews in the area (Adan et al., 2017; Murray et al., 2017; Radhakrishnan et al., 2014; Sami et al., 2015). In total, 70 articles were identified for the final review.

Data extracted from all selected articles included: (a) measure of interest; (b) numbers in psychosis cannabis-using and non-using groups; (c) numbers in control cannabis-using and non-using groups; (d) whether longitudinal or cross-sectional study; (e) psychosis population of interest (whether early psychosis, chronic psychosis, diagnosis of interest, or whether high-risk cohort); (f) relevant findings. To assess risk of bias the following were extracted: (a) definition of cannabis use; (b) limitations of article; and (c) confounders considered in the study design – namely alcohol, tobacco, other recreational substance misuse and exposure to medications for psychosis. If data were not available from the main manuscript, supplementary data were searched. A confounder was noted as having been considered in any of the following instances: (a) the said confounder had been excluded at the inclusion/exclusion stage; *or* (b) as demonstrated in the data provided there were no statistically significant baseline differences between P-C (group with psychosis or high risk of psychosis who use cannabis) and P-NC (group with psychosis or high risk of psychosis who do not use cannabis) for the given confounder; *or* (c) the confounder was adjusted for statistically (for example using regression methods); *or* (d) the confounder was demonstrated not to have an effect on the measure of interest; *or* (e) a sensitivity analysis was undertaken to consider the effect of the results when the confounder of interest was removed. Because of the heterogeneity of outcome measures, this systematic review represents a qualitative synthesis of the data and meta-analysis was not undertaken.

#### Results

Of the 70 articles extracted (numbers of patients given are approximate as we have tried to account for overlapping cohorts), eight studies considered structural magnetic resonance imaging (MRI) studies in high-risk states for psychosis (P-C *n*=261; P-NC *n*=539); 16 considered structural MRI studies in the first 5 years of psychosis (P-C *n*=378; P-NC *n*=383); eight considered structural findings after the first 5 years (P-C *n*=131; P-NC *n*=116); six considered functional MRI findings (P-C *n*>55; P-NC *n*>36); 14 considered other neurobiological measures including peripheral findings and electrophysiology (P-C *n*=208; P-NC *n*=378); 12 considered the endocannabinoid system (P-C *n*=152; P-NC *n*=287); five considered the dopamine system (P-C *n*=44 ; P-NC *n*=59); three studies to date have investigated the glutamate system (P-C *n*=40; P-NC *n*=65); and two studies have investigated the GABAergic system (P-C *n*=24; P-NC *n*=28). Collectively, therefore, this systematic review collates data for over 1253 participants for P-C and 1843 participants for P-NC (including both patients and those at high risk). The results of the complete systematic review can be seen in Tables 2 and 3. Below we discuss the salient features. Where relevant, in the discussion that follows additional studies have been cited for contextual purposes.

**Table 2.** Characteristics of included studies in patients/risk of psychosis comparing cannabis users (P-C) with non-users (P-NC): Neurobiological differences.

Study	Method	Measure	Population	Cannabis use definition	*N*				Findings	Limitations	Confounders considered:			
					P-C	P-NC	HC-C	HC-NC			ETOH	Other drug	AP	Tob
***(a) MRI Studies in high-risk states***														
Welch et al. (2011a)	MRI - **2 years longitudinal**	Thalamus & mediotemporal volumes	High genetic risk of schizophrenia	Cannabis use during intervening 2 years between scans	25	32			Bilateral thalamic volume loss P-C vs. P-NC. Amygdala-Hippocampal volume change non-significant	No HC comparator group. Unclear how many progressed to psychotic disorder	Y	Y	N/A	Y
Welch et al. (2011b)	MRI (cross-sectional)	Volumetric analysis of ventricles, prefrontal lobe, amygdala-hippocampal complex and thalamic nuclei	High genetic risk of schizophrenia	P-C: Regular use; P-NC: Use of ≤3 occasions/lifetime	73	69	15	20	Cannabis use correlated with increased ventricular size (particularly 3rd ventricle) in risk group. This relationship does not hold in control group	OR of progression to psychotic disorder 3.18, P-C vs. P-NC. Unclear whether this correlates with imaging findings	Y	Y	N/A	Y
Habets et al. (2011)	*Discussed below (see (a)(ii))*													
Stone et al. (2012)	MRI (cross-sectional)	Regional grey matter (GM)	At-risk mental state (ARMS)	Ever use. Analysis undertaken by cannabis intake (occasions per year)	19	8	14	13	Heavier cannabis use associated with reduced GM volume in prefrontal cortex. No distinct effects in ARMs vs. HCs	Small study. Does not establish functional correlate of GM loss	Y	Y	Y	Y
Welch et al. (2013)	MRI tensor-based morphometry, **2 years longitudinal**	Regional grey matter (GM)	High genetic risk of schizophrenia	Cannabis use during intervening 2 years between scans	23	32			Reduction in right anterior hippocampus and left superior frontal gyrus P-C vs. P-NC	No HC comparator group. Unclear how many progressed to psychotic disorder	Y	Y	N/A	Y
Rapp et al. (2013)	*Discussed below (see (a)(ii))*													
Buchy et al. (2015)	Resting state fMRI functional connectivity (cross-sectional)	Thalamic functional connectivity	Clinical high-risk (NAPLS-2 cohort)	Rated by severity of use, frequency of use, age of first use and whether early or late onset (cut off age 15)	50	112	92	13	Significant correlation between age of onset of cannabis use in CHR and thalamic hyper-connectivity with sensory motor cortex (significant on left, trend level on right)	Unclear how many progress to psychotic disorder	Y	N	N	Y
Buchy et al. (2016)	MRI (cross-sectional)	Hippocampus, amygdala, thalamus volumes	Clinical high-risk (NAPLS-2 cohort)	Use in the last month	132	387		204	No difference P-C vs. P-NC after adjustment for tobacco and alcohol	No metric for lifetime/cumulative exposure. No HC-C group	Y	N	Y	Y
***(b) Structural MRI studies in patients (within 5 years of first episode)***														
Cahn et al. (2004)	MRI (cross-sectional)	Regional brain volumes	Recent-onset schizophrenia, schizoaffective, schizophreniform	DSM IV cannabis abuse/dependence	27	20			No difference total brain volume, caudate, cerebellar volume. Decreased left/right asymmetry P-C vs. P-NC	P-C significantly younger than P-NC (21.13 years vs. 27.61)	N	Y	Y	N
Szeszko et al. (2007)	MRI (cross-sectional)	Prefrontal brain volumes	Recent-onset schizophrenia, schizoaffective, schizophreniform	DSM IV cannabis abuse/dependence	20	31		56	P-C significantly decreased anterior cingulate grey matter vs. P-NC	Manual outlining of frontal brain regions	Y	Y	Y	N
Wobruck et al. (2009)	MRI (cross-sectional)	Volumetric measure of superior temporal gyrus, amygdala-hippocampal complex, cingulum	Recent-onset schizophrenia, schizoaffective disorder recently admitted to hospital	Lifetime abuse	20	21			P-C vs. P-NC: no effect on brain morphology between groups	P-C all used cannabis, also reported: stimulants (35%), opiates (10%), cocaine (40%), hallucinogens (5%), alcohol (10%)	N	N	Y	N
Bangalore et al. (2008)	MRI (cross-sectional)	Dorsolateral prefrontal cortex, hippocampus, posterior cingulate, cerebellum, intracranial volume	Schizophrenia, schizoaffective, schizophreniform	Lifetime use vs. never use	15	24		42	Right posterior cingulate showed trend for reduced GM volume P-C vs. P-NC. No difference for DLPFC, cerebellum, hippocampus, whole brain volume	8/15 (53%) P-C group had poly-substance use	N	Y	Y	N
Peters et al. (2009)	MRI – Diffusion tensor imaging (cross-sectional)	Fractional anisotropy (FA) & markers of white matter integrity	Recent-onset schizophrenia/schizoaffective/schizophreniform	P-C: Cannabis use < age of 17; P-NC No cannabis use < 17	24	11		21	FA higher in patients using before 17 than controls in anterior capsule, fasciculus uncinatus, frontal white matter	Small numbers. Requires further replication and correlation with function	Y	Y	Y	N
Dekker et al. (2010)	MRI: High resolution structural and diffusion tensor imaging (cross-sectional)	WM density, fractional anisotropy	DSM IV schizophrenia, male	At least weekly for 6 months of life. Early-Onset Cannabis use ≤15; Late Onset Cannabis Use≥17	18	8		10	Reduced FA and WM density in left posterior corpus collosum, right occipital lobe, left temporal lobe, for P-NC compared with early-onset users	Small groups size – 8 early-onset users and 10 late-onset users	N	N	Y	N
James et al. (2011)	MRI: voxel-based morphometry and diffusion tensor imaging (cross-sectional)	Regional brain volumes and fractional anisotropy. Neurocognitive IQ (measured by WASI)	Adolescents with DSM IV schizophrenia	More than 3 days/week for 6 months. All participants last use ≥28 days prior	16	16		28	P-C vs. P-NC widespread grey matter loss in mediotemporal, insular, cerebellum, occipital and ventral striatum areas. Decreased FA in brainstem, internal capsule, corona radiata, superior and inferior longitudinal fasciculus	Age of onset younger in P-NC vs. P-C - may represent atypicality of adolescent schizophrenia	N	Y	Y	N
Cohen et al. (2012)	MRI (cross-sectional)	Cerebellar grey and white matter volumes	First-episode schizophrenia	Juvenile cannabis use. Average age of first use (PC-C) 15.1, (HC-C) 15.5; lifetime doses (PC-C) 22700, (HC-C) 17900	6	13	17	19	Dose-related decreased GM in cerebellum for HC-C vs. HC-NC. Decreased GM changes in P vs. HC. No interaction of cannabis use with diagnosis on GM cerebellar changes	Small P-C group	N	Y	N	N
Kumra et al. (2012)	MRI (cross-sectional)	Volumes: frontal, temporal, parietal, subcortical, cortical thickness, surface area, cognitive measures	Early-onset schizophrenia	Lifetime cannabis abuse/dependence. Excluded positive urine test	13	35	16	51	PC-NC vs. HC-NC and HC-C vs. HC-NC decreased left superior parietal cortex, relative sparing in PC-C. PC-C vs. PC-NS: GM left thalamus volume reduced	Excluded current users	N	N	Y	N
Schnell et al. (2012)	MRI (cross-sectional)	Regional brain volumes & neurocognitive testing	DSM IV schizophrenia	Lifetime cannabis abuse/dependence	30	24			P-C vs. P-NC higher GM density in left middle frontal gyrus. Significantly correlated with Continuous Performance Task (indexes working memory and attention, *r*=0.681)	Needs further replication	Y	Y	Y	Y
Cunha et al. (2013)	MRI (cross-sectional)	Grey matter and lateral ventricle volumes & neurocognition (WMAT, COWAT, digit span)	First-episode psychosis	Lifetime history of at least 3 times per month for 1 year	28	78		80	P-NC vs. HC-NC decreased grey matter but in P-C vs. P-NC less grey matter loss in left middle frontal gyrus, left hippocampus, left parahippocampal gyrus. P-C vs. P-NC fewer attentional and executive deficits	P-C younger and more time in full time education than P-NC	Y	Y	Y	N
Haller et al. (2013)	MRI: voxel-based morphometry and diffusion tensor imaging & neurocognitive testing (TAP, WMS-R) (cross-sectional)	Grey matter volume & tract-based spatial statistics & fractional anisotropy	First-episode psychosis	1. Heavy Use: near daily use for at least 1 year prior to presentation; 2. Light Use>lifetime use 10 times, less than heavy use; 3. Considered as non-user if used up to 10 times	33	17			No difference between P-C and P-NC on voxel-based morphometry or DTI analysis or neurocognitive measures. No difference between heavy vs. light use	Small size ?underpowered	N	Y	Y	N
Rapp et al. (2013)	MRI (cross-sectional)	Grey matter volumes in cingulate cortex	At-risk mental state and first-episode psychosis	Current cannabis use	Pts:8 ARMS:14	Pts:15 ARMS:22			Negative effect of cannabis use on posterior cingulate cortex and left anterior cingulate for both FEP and ARMS	Small sample size. Manual segmentation – high interrater reliability	Y	N	Y	N
Malchow et al. (2013)	MRI & MRS (cross-sectional)	Volumetric analysis: hippocampus, amygdala, caudate, putamen, thalamus, corpus callosum; MRS metabolites (N-acetyl-aspartate) indexes neuronal integrity	First-episode schizophrenia	Cannabis abuse	29	20		30	Psychosis patients volume loss left hippocampus and amygdala vs. controls. P-C vs. P-NC larger mid-sagittal area of corpus callosum. P-C had higher left putamen N-acetyl aspartate/choline	No functional correlate. Limited information on cannabis use	N	N	N	N
Epstein and Kumra (2015)	MRI **Longitudinal - 18 month follow-up**	Cortical thinning & neurocognitive (D-KEFS Tower Test)	Early-onset schizophrenia/schizoaffective/schizophreniform	Lifetime cannabis abuse/dependence at baseline	11	17	17	34	No significant main effect for psychosis, but main effect for cannabis use disorder – widespread cortical thinning. No significant effect for cannabis use disorder × psychosis interaction	Small P-C group. Needs replication in a larger cohort	Y	N	N	Y
Koenders et al. (2015)	MRI (cross-sectional)	Surface-based analysis of a priori brain regions	DSM IV psychotic disorders (male patients only)	DSM IV cannabis abuse/dependence	80	33		84	P-C vs. P-NC: increased putamen enlargement in P-C. Patients vs. controls: smaller volumes amygdala, putamen, insula, parahippocampus, fusiform gyrus	Did not correct for smoking or medication	Y	Y	Y	Y
***(c) Structural MRI Studies in Patients (after 5 years of first episode)***														
Rais et al. (2008)	MRI – **5 years longitudinal**	Ventricle size and total grey matter (GM) volume change	Recent-onset schizophrenia	Ever cannabis use during scan interval (5 years)	19	32		31	Larger ventricular size and reduced GM in direction: P-C>P-NC>HC. P-C vs. P-NC less pronounced symptomatic improvement	No HC-C group to disentangle psychosis × cannabis interaction	Y	Y	Y	N
Rais et al. (2010)	MRI – **5 years longitudinal**	Cortical thickness	Recent-onset schizophrenia	Ever cannabis use during scan interval (5 years)	19	32		31	P-C vs. P-NC no baseline difference. Over 5 years increased cortical thinning in DLPFC, ACC and left occipital lobe	Does not establish functional correlate of cortical thinning	Y	Y	Y	N
Habets et al. (2011)	MRI (cross-sectional)	Cortical thickness	DSM IV psychotic disorders, siblings; controls	1 Subjects who have never used cannabis; 2 subjects who have used 1–39 times (moderate); 3 subjects who have used ≥40 times (heavy)	52 pts; 33 siblings	28 pts; 53 siblings	21	48	Patients with heavy use had lower cortical thickness than those with no use. Same relationship for siblings but not for controls	Needs replication in larger samples. No function correlate of diminished cortical thickness	N	Y	Y	N
Solowij et al. (2011)	MRI (cross-sectional)	Cerebellar grey matter and white matter volume	Schizophrenia, right-handed male	Long-term heavy cannabis use (near daily use for ≥9 years)	8	9	15	16	No group differences in GM volume. Direction of WM volume: HC-NC>P-NC>HC-C>P-C	Small groups P-C and P-NC	Y	Y	N	Y
Solowij et al. (2013)	MRI (cross-sectional)	Hippocampal shape analysis	Schizophrenia	Long-term regular use; >60000 doses last 10 years	8	9	15	16	Hippocampal shape changes in each group vs. HC-NC with greatest changes in P-C vs. H-C	Small P-C group. Chronic patient group. No functional correlation	Y	Y	N	Y
Smith et al. (2014)	MRI (cross-sectional)	Surface-based representations of globus pallidus (GP), striatum, thalamus and working memory	DSM IV Schizophrenia	DSM IV lifetime cannabis abuse/dependence	15	28	10	44	Morphological shape differences observed in cannabis groups (P-C vs. P-NC and HC-C vs. HC-NC) in striatum, GP and thalamus. Morphological changes more pronounced in P-C than HC-C. For both P-C and HC-C striatal and thalamic changes correlated with WM deficits and younger age of CUD diagnosis	Cannabis use group not had substance misuse diagnosis in 6 months prior to the study. Cross-sectional – need longitudinal follow-up	Y	Y	Y	Y
Smith et al. (2015)	MRI (cross-sectional)	Surface-based analysis of hippocampus, episodic memory (logical memory II test)	DSM IV schizophrenia	DSM IV cannabis abuse/dependence 6 months previously	15	28	10	44	Effect of shape changes by cannabis use disorder ‘cannabis-like shape’. Separate changes on shape of hippocampus by schizophrenia ‘schizophrenia-like shape’. P-C group demonstrated increasing cannabis shape changes with increasing duration of cannabis use disorder. P-C vs. P-NC trend level worse on episodic memory task	Prolonged period of abstinence for cannabis users (at least 6 months)	Y	Y	Y	Y
Rigucci et al. (2015)	MRI – diffusion tensor imaging (cross-sectional)	Fractional anisotropy & markers of white matter integrity	First-episode psychosis (ICD-10 diagnosis confirmed using OPCRIT+)	Lifetime history of cannabis use. Further analyses undertaken on other parameters (age of first use, potency of use, frequency of use)	37	19	22	21	Higher potency cannabis associated with disturbed corpus callosum microstructure in both patients with psychosis and cannabis users	No functional correlate described	N	N	N	N
***(d) functional MRI studies in patients***														
Loberg et al. (2012)	fMRI – dichotic auditory perception task	BOLD activation of default mode network and effort mode network	DSM IV schizophrenia	Lifetime cannabis use. Current users excluded	13	13			P-C vs. P-NC: increased activation in regions involved in effort mode network and decreased activation in default mode network	Unconventional method of indexing network activity. Cannot extend work to current users	N	N	Y	N
Bourque et al. (2013)	fMRI – emotional memory task	BOLD activation in emotional picture recognition	DSM IV schizophrenia, males	Cannabis use disorder (abuse/dependence) diagnosed in last 6 months	14	14		21	P-C vs. P-NC: medial prefrontal cortex activation increased in emotional picture recognition	Small study. Needs further replication in larger samples	N	Y	Y	N
Potvin et al. (2013) (from same group as Bourque et al (2013)?same patient group)	fMRI – visuospatial task and mental rotation	Regional BOLD activation	DSM IV schizophrenia, males	Cannabis use disorder (abuse/dependence) diagnosed in last 6 months	14	14		21	P-C vs. P-NC: preserved activation left superior parietal gyrus (decreased in P-NC). No difference in task performance P-C vs. P-NC	Small study. Needs further replication in larger samples	N	Y	Y	N
Machielson et al. (2014)	fMRI attentional bias using a classical Stroop task and cannabis Stroop task after 4 weeks of treatment with risperidone or clozapine	Stroop task performance and BOLD activation. Classical Stroop indexes selective attention, Cannabis stroop to index attentional bias	DSM IV schizophrenia, schizoaffective, schizophreniform disorder, male gender	Cannabis abuse/dependence	28	8		19	No difference P-C vs. P-NC in performance in Stroop task. P-C vs. P-NC no difference in regional activation for classical Stroop. Greater activation in left and right amygdala for P-C vs. P-NC	Small P-NC group ?underpowered. Also main purpose of study not to compare P-C vs P-NC groups but to compare risperidone vs. clozapine. Confounders not adjusted for outcomes of interest	N	N	N	N
Peeters et al. (2015)	Resting state fMRI functional connectivity (cross-sectional)	Dorsolateral prefrontal cortex functional connectivity (DLPFC-fc) & neurocognitive testing (WAIS III and others)	Psychosis, unaffected siblings and controls	Ever use vs. never use	Patients with psychosis (*n*=73); Unaffected siblings (*n*=83); Controls (*n*=72); Numbers not clearly stated by cannabis use	No significant group × cannabis interaction for DLFPC-fc and no significant interaction with neurocognitive testing	Most patients on medication. Effect of this unclear. Use of current use maybe more sensitive to change than lifetime cannabis use	N	N	Y	N			
Machielson et al. (2017)	Cue reactivity to cannabis and neutral images fMRI	Regional BOLD activation to cannabis images	DSM IV schizophrenia/schizoaffective/schizophreniform	Lifetime cannabis use disorder (based on CIDI)	30	8		20	P-C vs. P-NC greater activation to cannabis images in right amygdala and left and right thalamus. *Also* 4 weeks of clozapine superior to risperidone in reducing craving in P-C	Designed to compare clozapine vs. risperidone rather than to compare P-C vs. P-NC. Small P-NC group	N	N	N	N
***(e) Other neurobiological measures***														
Jockers-Scherubl et al. (2003)	Blood (cross-sectional)	Serum nerve growth factor (NGF) (cross-sectional)	DSM IV schizophrenia, presenting as inpatient, medication naïve	>0.5 g on average per day for at least 2 years. Positive UDS excluded	21	76	11	61	P-C NGF levels mean=412.9; P-NC 26.3; HC-C 20.1; HC-NC 33.1. Significantly raised in P-C group	Small numbers in cannabis-using group. P-C onset of psychosis younger than P-NC	Y	Y	Y	N
Jockers-Scherubl et al. (2004)	Blood (cross-sectional)	Serum brain derived neurotrophic factor (BDNF) (cross-sectional)	DSM IV schizophrenia, medication naïve	>0.5g on average per day for at least 2 years. Positive UDS excluded	35	102	11	61	P-C BDNF levels mean=17.7; P-NC 13.1; HC-C 13.1; HC-NC 13.2. Highest in P-C group, significant vs. H-C and P-NC	P-C onset of psychosis younger than P-NC. Needs replication	Y	Y	Y	N
Jockers-Scherubl et al. (2006)	Blood (including follow-up of Jockers-Scherubl et al. (2003))	Serum nerve growth factor (NGF) (cross-sectional)	DSM IV schizophrenia, treated with medication for psychosis for 4 weeks	>0.5 g on average per day for at least 2 years. Positive UDS excluded	42	66	24	51	No statistically significant difference between groups (i.e. NGF levels raised in P-C had normalized). Decrease across patient groups in NGF levels from Jockers-Scherubel et al. (2003). Also small cohort replicated Jockers-Scherubel et al. (2003) baseline results	Needs replication	Y	Y	Y	N
Benson et al. (2007)	Eye movement tracking (cross-sectional)	Fixation clustering, saccadic gaze	DSM IV cannabis-induced psychosis (CIP) vs. first-episode schizophrenia based on SCID	Based on diagnosis of CIP (see limitations)	6	11		22	Differences noted in visual scan paths. More restricted visual features in P-C patient with increased fixation clustering such that P-C>P-NC>HC-C. Alterations in saccadic gaze (frequency, amplitude, velocity) for P-C vs. HC-NC in altered pattern compared with P-NC vs. HC-NC	Small study and needs replication. Definition of P-C and P-NC in study based on CIP vs. schizophrenia. Three patients with schizophrenia (in P-NC) had used cannabis before developing schizophrenia	N	N	N	N
Rentzsch et al. (2007)	EEG with auditory stimuli (cross-sectional)	P50 Sensory gating	DSM IV schizophrenia/schizoaffective	History of chronic cannabis abuse. All users abstinent ≥28 days	15	12	11	18	No difference between P-C and P-NC. In HC-C P50 sensory deficit correlated with number of years with daily consumption. Relationship not present in other groups	Small sample size ?underpowered. There is a difference of circa 10% between P-C vs. P-NC but not significant	Y	Y	N	N
Rentzsch et al. (2011)	EEG with auditory stimuli (cross-sectional)	Mismatch negativity (MMN)	DSM IV schizophrenia/schizoaffective	Chronic cannabis use: at least 5 days per week for at least 1 year by self-report. All users abstinent ≥28 days	27	26	32	34	Frequency MMN amplitude P-NC<P-C<HC-C<HC-NC	No functional correlation shown. Requires replication	Y	Y	Y	Y
Scholes-Balog and Martin-Iverson (2011)	Electrophysiology with auditory stimuli (cross-sectional)	Prepulse Inhibition (PPI)	Schizophrenia/schizoaffective	Lifetime use	20	44	34	32	Alterations of PPI in all groups vs. HC-C such that reduced PPI in P-NC vs. HC-NC but this is diminished in P-C vs. HC-NC. Authors suggest cannabis may have medicating effect on impaired attentional modulation for patients and have similar effect on attentional modulation for patients and healthy controls	Preponderance of other substance abuse/dependence in last 12 months in P-C group (60%)	N	N	Y	Y
Pesa et al. (2012)	EEG with auditory stimuli (cross-sectional)	Mismatch Negativity (MMN) and P3a latency	Early psychosis, DSM IV psychotic disorders	History of past or current cannabis use at least monthly for one year	22	22		21	For MMN amplitude: P-NC<P-C<HC-NC. For MMN latency: P-C>P-NC and HC-NC. For P3a amplitude at frontal electrode: P-C<P-NC and latency P-C>P-NC	No functional correlate identified	Y	Y	N	N
Van Tricht et al. (2013)	EEG with auditory oddball paradigm (cross-sectional)	N100, N200, P200, P300	Ultra-high risk	History of use ≥5 times/lifetime. Use in last month	19	29	21	29	No significant differences between P-C and P-NC	Gender differences between groups (18% female P-C; 45% female P-NC). Although tobacco use was adjusted for, other recreational drug use not accounted for	Y	Y	Y	N
Cassidy et al. (2014)	Electrophysiology – EEG and facial electromyography to visual stimuli of natural and cannabis rewards (cross-sectional)	Late positive potential (LPP), facial electromyography, skin conductance. Follow-up after 1 month for cannabis usage	DSM IV schizophrenia/schizoaffective	Active cannabis use disorder in past 1 month vs. no cannabis use in last 3 months (P-NC)	20	15	20	15	P-C show blunted responses to natural rewards but spared response to cannabis stimulus. LPP in P-C predicts cannabis usage at 1 month	Cannabis images prepared specifically for this task and not validated in previous tasks	N	Y	Y	Y
Hagenmuller et al. (2014)	EEG with somatosensory evoked potential (cross-sectional)	N20, P25	Ultra-high risk (UHR) assessed by structured interview for prodromal symptoms and high risk (HR) for schizophrenia assessed by schizophrenia proneness interview		HR 13; UHR 12	HR 36; UHR 61	3	42	P-C vs. P-NC: in both HR and UHR groups cannabis users showed higher N20-P25 source strength than non-users. Not calculated in HC due to small numbers of HC-C	Small cannabis arms. Possible effect of confounding variables (see right)	N	N	N	N
Winton-Brown et al. (2015)	Electrophysiology with auditory stimuli (cross-sectional)	Prepulse inhibition (PPI) and prepulse facilitation (PPF)	At-risk mental state	Urine drug sample positive	6	18	5	18	PPI: HC-C vs. HC-NC increased PPI, P-C vs. P-NC decreased PPI; PPF: No group × substance use interaction	Small numbers of cannabis participants. UDS only indicates recency of use	N	Y	Y	Y
Morales-Munoz et al. (2015)	Electrophysiology with auditory stimuli (cross-sectional)	Prepulse inhibition (PPI)	First-episode psychosis, on treatment (assessed by SCID for DSM IV)	Lifetime cannabis abuse/dependence (assessed by SCID), currently abstinent	21	14		22	PPI: at 30 ms reduced PPI for P-C vs. HC-NC and P-NC vs. HC-NC. At 60 ms reduced PPI for P-NC vs. HC-NC but not for P-C vs. HC-NC. At 120 ms no difference between groups	No assessment of alcohol or other drug use. Although all had been on pharmacotherapy cumulative doses not matched	N	N	Y	Y
Rentzsch et al. (2016) *(some overlap of participants with* Rentzsch et al., 2007)	EEG to auditory novelty and oddball paradigms (cross-sectional)	P300	Schizophrenia	Chronic cannabis use: at least 5 days per week for at least 1 year by self-report. All users abstinent ≥28 days	20	20	20	20	Different effects of cannabis use on patients and controls. Unadjusted: Early novelty P300 HC-NC>HC-C>P-NC>P-C. Late novelty P300 HC-NC>HC-C>PC-NC~–P-C. Parietal oddball HC-NC>HC-C>P-NC>P-C	No significant diagnosis interaction for early novelty p300 and oddball paradigm remained when nicotine and alcohol use entered as covariates. Significant difference between illicit drug use between groups	Y	N	Y	Y

*(a) MRI studies in high-risk groups (genetic, familial, clinical high risk)*.

*(b) MRI structural in patients with psychosis (including volumetric, morphometry and shape analysis, diffusion tensor imaging) first 5 years*.

*(c) MRI structural in patients with psychosis (including volumetric, morphometry and shape analysis, diffusion tensor imaging) after 5 years*.

*(d) Functional MRI studies in patients with psychosis*.

*(e) Other neurobiological measures (EEG, eye-tracking)*.

P-C: Psychosis/at-risk patients with cannabis use; P-NC: Psychosis/at-risk patients without cannabis use; HC-C: Non-psychosis controls with cannabis use; HC-NC: Non-psychosis controls without cannabis use; ETOH: Alcohol; AP: medication for psychosis; Tob: Tobacco.

**Table 3.** Characteristics of included studies in patients/risk of psychosis comparing cannabis users (P-C) with non-users (P-NC): Neurochemical differences.

Study	Method	Measure	Population	Cannabis use definition	*N*				Findings	Limitations	Confounders considered:			
					P-C	P-NC	HC-C	HC-NC			ETOH	Other drug	AP	Tob
***(A) Endocannabinoid system***														
Dean et al. (2001)	Post-mortem autoradiography	[3H]CP-55940 to index CB1 receptor density at dorsolateral prefrontal cortex, caudate-putamen and temporal lobe regions	Schizophrenia assessed after psychologist and psychiatrist case notes review using diagnostic instrument for brain studies (DIBS)	THC in blood at time of death	5	9	4	9	Increased DLPFC binding in patients vs. controls. Increased binding in caudate-putamen in cannabis groups independent of whether patients or controls	Small numbers. Did not adjust for key confounders (see right)	N	N	N	N
Zavitsanu et al. (2004)	Post-mortem autoradiography	[3H]SR141716A to index CB1 receptor density binding at anterior cingulate cortex	DSM III/DSM IV schizophrenia using case notes review using SCAN and DIBS	Lifetime ever use	5	5		9	No difference P-C vs. P-NC. Patients increased CB1 receptor binding compared with controls	Small group size	N	N	Y	N
Monterrubio et al. (2006)	Peripheral blood (cross-sectional)	Fatty acid levels in blood	Schizophrenia treated with clozapine	Ever used. Last use ≥6 months ago	6	6			In cannabis users only: arachadonic acid correlated with total fatty acid. Linoliec acid correlated with stress	Small size; Discontinued users	N	Y	Y	N
Leweke et al. (2007)	Cerebrospinal fluid (CSF) (cross-sectional)	Anandamide levels	Schizophrenia	High frequency in cannabis group: ≥20 times per life. Low frequency in non-cannabis group: ≤5 times per life	22	25	26	55	P-NC markedly higher anandamide in CSF than P-C. No difference between HC and HC-NC	Needs replication	N	N	Y	N
Deng et al. (2007)	Post-mortem autoradiography	[3H]SR141716A and [3H]CP-55940 both to index CB1 receptor density at superior temporal gyrus	DSM IV schizophrenia using case notes review using SCAN and DIBS	Not clear	4	4	8 non-psychiatric controls – unclear if any cannabis use history		P-C vs. P-NC no difference between groups. No difference between patients vs. controls	Small numbers. Insufficiently clear about cannabis use history	N	N	Y	N
Eggan et al. (2008)	Post-mortem immunocytochemistry	CB1 receptor mRNA in dorsolateral prefrontal cortex (Brodmann area 9)	Schizophrenia/ schizoaffective	Not clear	7	16		23	No difference between P-C and P-NC. Reduction of around 10–15% in CB1 receptor transcript expression in patients vs. controls	Study not designed to determine difference of P-C vs. P-NC. Tested as a possible confounding variable	N	Y	Y	N
Eggan et al. (2010) *Includes 14 patients and 14 HCs from* Eggan et al. (2008)	Post-mortem immunocytochemistry	CB1 receptor protein expression in dorsolateral prefrontal cortex (Brodmann area 46)	DSM IV Schizophrenia/schizoaffective using case notes review and structured interview with relative. Controls: Controls with no psychiatric history (NP) and depressed (DEP)	Lifetime history of cannabis use	6	15	0 NP, 3 DEP	26 NP, 7 DEP	P-C vs. P-NC no significant difference between groups. Reduction of around 19–23% in CB1 receptor density in patients with psychosis vs. controls	Study not designed to determine difference of P-C vs. P-NC. Tested as a possible confounding variable	N	Y	Y	N
Ho et al. (2011)	Structural MRI, cognitive assessment (WAIS subscales) by CB1 receptor genotype and cannabis interaction (cross-sectional)	White matter & WAIS subscales	Schizophrenia patients, P-C arranged by CB1 receptor genotype (12 tagged SNPs)	Cannabis abuse or dependence	52	183			Three CB1 receptor polymorphisms associated with decreased WM volume. One also associated with decreased processing speed and attention in P-C	Needs replication in larger cohort. Possible confounding from other substance use	Y	Y	Y	N
Onwuameze et al. (2013) *(from same sample as* Ho et al. (2011))	Structural MRI by MAPK14 genotype and CB1 receptor and cannabis interaction (cross-sectional)	White matter	Schizophrenia patients, P-C arranged by MAPK14 Receptor genotype (nine tagged SNPs)	Cannabis abuse or dependence	52	183			MAPK14 and CB1 receptor specific alleles associated with small white matter brain volume in heavy cannabis use. Independent and additive effect	As Ho et al. (2011). Also no functional correlate of WM loss demonstrated	Y	Y	Y	N
Ceccarini et al. (2013)	PET (cross-sectional)	[18F]MK-9470 mSUV (indexes CB1 receptor)	Schizophrenia	Ever use. Last use for all participants ≥6 months ago	35	32		12	Patients with a history of heavy cannabis use no significant difference in binding vs. medium, low or never use	Not designed to test P-C vs P-NC	N	N	N	N
Volk et al. (2014) *(same participant group as* Eggan et al. (2008)	Post-mortem autoradiography	[11C]OMAR binding (indexes CB1 receptor)	Schizophrenia/ schizoaffective	Not clear	7	14		21	[11C]OMAR binding did not differ between P-C vs. P-NC	Study not designed to determine difference of P-C vs. P-NC. Tested as a possible confounding variable	N	N	N	N
Ranganathan et al. (2016)	PET (cross-sectional)	[11C]OMAR VT (indexes CB1 receptor)	Male Schizophrenia	Ever use. Lifetime cannabis use disorder excluded. 1/25 patients with recent use	16	7	Total 18 HC. Lifetime use unclear	No significant correlations between cannabis use and VT	Not designed to test P-C vs. P-NC	N	N	N	N	
***(b) Dopamine system***														
Dean et al. (2003)	Post-mortem autoradiography	[3H]Mazidol for DAT; Tyrosine Hydroxylase	Schizophrenia	Blood test +ve	5	9	4	10	No significant difference between CBS users and non-users	Small sample. Limited cannabis information	N	N	Y	N
Bowers and Kantrowitz (2007)	Peripheral blood (cross-sectional)	Plasma homovanillic acid	Inpatient FEP vs. inpatient non-psychosis	Urine test +ve	5	15		18	P-C group elevated HVA levels vs. P-NC and others (*p*=0.001)	Small sample. Limited cannabis information	N	N	N	N
Safont et al. (2011)	SPECT (cross-sectional)	[123I]IBZM striatal/frontal ratio to index D2/D3 receptor availability	Untreated FEP patients	Use 3 units/day for last 3 months (*n*=14)	14	23		18	No significant difference between P-C and P-NC	Used S/F ratio but frontal binding may be altered in cannabis use	N	Y	Y	N
Kuepper et al. (2013)	PET: 8 mg inhaled THC vs. placebo Acute challenge on dopamine function (interventional)	[18F] Fallypride displaced	Psychosis patients, first-degree relatives of patients, healthy controls	Self-report ever use	8 pts 7 rel’s		9		THC induced significant striatal displacement of fallypride in patients and relatives but not controls	No comparison between P-C and P-NC	Y	Y	Y	Y
Mizrahi et al. (2014)	PET: stress task to induce dopamine release (cross-sectional)	[11C]PHNO displaced	Clinical high risk	Use at least 3 times/week	12	12			Decreased displacement in P-C group and increased in P-NC group in striatum	Unable to determine whether blunted dopamine release is marker of cannabis use or addiction	N	Y	Y	Y
(c) Glutamate system														
Rentzsch et al. (2011)	See Table 2 (e) (Mismatch Negativity believed to index glutamatergic function via NMDA receptor)													
Pesa et al. (2012)	*See Table 2 (e) (Mismatch Negativity believed to index glutamatergic function via NMDA receptor)*													
Rigucci et al. (2017)	Magnetic resonance spectroscopy (cross-sectional)	Glutamate medial prefrontal cortex & neurocognitive assessment (MATRICS battery)	Early psychosis and healthy controls	Current cannabis use	18	17		33	Decreased glutamate in P-C vs. P-NC. Impaired working memory P-C vs. P-NC	Neuro-cognitive impairment in P-C rather than sparing. ?atypical sample. Requires further replication	Y	N	Y	Y
***(d) GABAergic system (indexed through cortical inhibition)***														
Wobrock et al. (2010)	Transcranial Magnetic Stimulation (cross-sectional)	Short-interval cortical inhibition (SICI), intracortical facilitation (ICF)	First-episode schizophrenia	P-C: lifetime use of ≥20 times per lifetime; P-NC Lifetime use of ≤5 times	12	17			Reduced SICI and enhanced ICF for P-C vs. P-NC indicating GABAergic deficit and intracortical disconnectivity	SICI and ICF not direct measures of GABA-A. No comparison with HC groups	N	Y	Y	N
Goodman et al. (2017)	Transcranial magnetic stimulation (cross-sectional)	SICI/ICF	Schizophrenia/schizoaffective disorder	DSM IV cannabis dependence	12	11	10	13	Increased SICI for PC vs. P-NC indicating increased GABA-A mediated inhibition, reduced SICI HC-C vs. HC-NC. No significant difference for ICF	SICI and ICF not direct measures of GABA-A	Y	Y	Y	N

*(a) Endocannabinoid System*.

*(b) Dopamine System*.

*(c) Glutamate System*.

*(d) GABAergic System*.

P-C: Psychosis/at-risk patients with cannabis use; P-NC: Psychosis/at-risk patients without cannabis use; HC-C: Non-psychosis controls with cannabis use; HC-NC: Non-psychosis controls without cannabis use; ETOH: Alcohol; AP: medication for psychosis; Tob: Tobacco.

#### Evidence from studies in electrophysiology and other neurobiological measures

In a series of early studies, Jockers-Scherubl and colleagues demonstrated markedly elevated neurotrophins in untreated cannabis-using patients with psychosis compared with both non-using patients and cannabis-using controls: serum nerve growth factor (NGF) and brain derived neurotrophic factor on initial admission (Jockers-Scherübl et al., 2003, 2004). Baseline NGF elevation was replicated in a follow-up study, and furthermore the elevation was noted to normalize following 4 weeks on medication treatment for psychosis (Jockers-Scherübl et al., 2006). These studies raise the possibility of distinct neuronal damage in the cannabis-using psychosis group, with neurotrophins as a state-dependent marker, although these results were not specific only to cannabis, as a mixed group of poly-substance misuse also demonstrated elevated neurotrophin levels. Although intriguing, these studies do not necessarily implicate neurotrophin elevation in the pathway of psychotic disorder. Of note there were small numbers of cannabis controls, and it is possible that elevated levels rescinded after decrease in use after inpatient admission rather than treatment.

A number of distinct electrophysiological alterations have also been noted in P-C versus P-NC indicating pre-attentional, attentional and reward cue deficits. Although one study demonstrated no differences between cannabis-using and non-using patients, in P50 sensory gating (Rentzsch et al., 2007), differences have been demonstrated in prepulse inhibition (PPI) with relative sparing of the diminished PPI amplitude seen in P-NC; (Morales-Muñoz et al., 2015; Scholes-Balog and Martin-Iverson, 2011); reduced P300 amplitudes for P-C versus P-NC to novelty and oddball auditory stimuli (Rentzsch et al., 2016), modulation of mismatch negativity (MMN) (Pesa et al., 2012; Rentzsch et al., 2011) and altered late positive potential to reward cues such that there appears to be blunting of response to natural but not cannabis visual cues in the P-C group (Cassidy et al., 2014). MMN is believed to index glutamatergic function and is discussed further below (see III(iii)). Taken together, these indicate neurophysiological abnormalities in the P-C group neither accounted for fully by psychosis (P-NC group) or cannabis use (HC-C group). There appears to be some evidence for modulation in clinical high-risk groups of PPI: one study has paradoxically shown that PPI is increased in cannabis use verses healthy controls but decreased in at-risk mental state subjects (Winton-Brown et al., 2015). Other studies have failed to elicit differences between P-C and P-NC in somatosensory evoked N20, P25 (Hagenmuller et al., 2014), auditory oddball N100, N200, P200 and P300 (Van Tricht et al., 2013).

One further body of work has found PPI deficits in cannabis-induced psychotic disorder intermediate between healthy controls and those of schizophrenia with cannabis use. These studies were not included because of the lack of a P-NC group; however, they have been used to argue a neurobiological basis to a differentiation between cannabis-induced psychotic disorder (CIPD) and schizophrenia with cannabis use (Morales-Muñoz et al., 2014, 2017). Further evidence for this comes from a small study demonstrating restrictions in visual field and alterations in saccadic gaze from P-C versus controls as opposed to patients with schizophrenia versus controls (Benson et al., 2007). However, to date there is limited work to determine whether CIPD is a distinct entity to other psychotic disorders complicated by use of cannabis.

#### Neuroimaging approaches

*1. MRI in high-risk states*

Two main cohorts have reported on structural MRI measures in those at high risk who use cannabis and who do not. The Edinburgh High Risk Study recruited first-degree family members of patients. Cross-sectional and longitudinal analysis demonstrated cannabis to be associated with loss of brain volume and subcortical structures including thalamic, mediotemporal and frontal areas (Welch et al., 2011a, b, 2013). These were a non-clinical cohort (i.e. non-treatment seeking) and with limited healthy control cannabis users it is not possible to disentangle the effects of psychosis liability from cannabis-induced changes which would be expected in the general population. A number of smaller studies in high-risk samples have supported regional grey matter loss associated with heavier cannabis use (cingulate cortex, prefrontal cortex, cortical thickness) (Habets et al., 2011; Rapp et al., 2013; Stone et al., 2012). In the largest cohort – the North American Prodrome Longitudinal Study (NAPLS) – evaluating clinical high-risk groups there was noted to be no difference between cannabis users (defined as use in the last month) in hippocampal, amygdala, and thalamic volumes and thalamic connectivity, although there was a correlation between younger age of onset and thalamic connectivity with the sensory motor cortex (Buchy et al., 2015, 2016).

*2. Structural MRI in the first 5 years of psychotic disorder*

There also appears to be differences detectable by neuroimaging approaches between cannabis-using and non-using patients in the first 5 years of psychotic disorder. Broadly speaking, given that both psychosis and cannabis use are known to be associated with atrophic changes and functional deficits compared with healthy non-using controls, there could alternative explanations to neurobiological differences between patients who use cannabis and those who do not: (a) there could be no difference between the groups (the null hypothesis); (b) there could be evidence of increased deficit or impairment in the P-C group compared with the P-NC group which may be accounted for by deleterious effects of cannabis use (i.e. an additive interaction of cannabis × psychosis); (c) there could be decreased deficit or impairment in the P-C group compared with the P-NC group (i.e. a sparing interaction of cannabis × psychosis).

In line with the null hypothesis, some studies have failed to find structural differences between groups (Cahn et al., 2004; Haller et al., 2013; Wobrock et al., 2009). Although these studies have their limitations of small sample sizes hence possibly being underpowered, they are no less than others. Others have found increased impairment in cannabis-using patients: increased grey matter loss in the cingulum (Bangalore et al., 2008; Rapp et al., 2013; Szeszko et al., 2007) and cerebellum (Cohen et al., 2012); whereas younger age of onset is associated with white and grey matter changes (James et al., 2011; Peters et al., 2009) and disruption of cortical maturation patterns (Epstein and Kumra, 2015).

As against those, other studies have reported fewer grey matter (Schnell et al., 2012) and white matter deficits (Dekker et al., 2010) in patients who use cannabis, or with mixed findings (Kumra et al., 2012). Some larger, more recent studies have similarly found evidence that P-C may have an altered neurobiological profile in early psychosis, not simply explained by the deleterious effects of cannabis use. Cunha et al. undertook MRI imaging in 28 cannabis-using patients with FEP, 78 non-cannabis-using patients with FEP and 80 healthy controls. Users were significantly younger than non-users, more likely to be male and better educated. There was preservation of grey matter volumes in medial temporal areas and the prefrontal cortex in cannabis users as opposed to controls (Cunha et al., 2013). Increased hippocampal volume in cannabis-using patients versus non-using patients was also demonstrated by Malchow and colleagues in a series of 47 FEP patients, 20 of whom were cannabis users and 30 healthy controls. Again, the cannabis-using group was more likely to be male and significantly younger. They were prescribed significantly more medication for psychosis at the time of scan, although cumulative exposure was matched (Malchow et al., 2013). A further study of 113 male age-matched FEP patients (80 cannabis users), and 80 healthy controls, showed the cannabis-using group to have parahippocampal (but not hippocampal), amygdyla and putamen enlargement (Koenders et al., 2015).

Collectively, there are varying findings from neuroimaging studies in early psychosis. The majority of studies indicate there are structural brain differences between cannabis-using patients and non-using patients. There are likely to be altered effects of cannabis on patients, with CB1 receptor-rich areas such as the cingulate particularly prone to atrophic change. As against this there is some evidence of sparing of brain regions such as mediotemporal or striatal regions. Which regions are affected or spared and the extent of these are likely to be a function of propensity to develop psychosis, patterns of cannabis use and duration and age of first use. Currently, the functional correlates and clinical significant of these brain differences remain to be fully elucidated.

*3. Structural MRI after the first 5 years of psychotic disorder*

In contrast to the conflicting evidence from studies in the early stages of psychosis, all studies after 5 years of psychotic disorder point towards degenerative changes in the cannabis-using patient group. These include two longitudinal studies of early psychosis followed up for 5 years (Rais et al., 2008, 2010). Alterations observed in the P-C group include: increased ventricular size (Rais et al., 2008), diminished cortical thickness (Habets et al., 2011; Rais et al., 2010), decreased grey (Rais et al., 2008) and white matter (Rigucci et al., 2015; Smith et al., 2014; Solowij et al., 2011) and cannabis-induced alterations in the hippocampus (Smith et al., 2015; Solowij et al., 2013), and change in surface shape of the striatum, globus pallidus and thalamus (Smith et al., 2014). Taken together, degenerative changes secondary to cannabis use have been consistently found in both cross-sectional and longitudinal designs. Although there is a lack of functional correlation in many of these studies, in that atrophy does not necessarily equate with loss of function, one may speculate in conjunction with the studies discussed in the first section that continued cannabis use in patients with established psychotic disorder (after the first 5 years) is both associated with impaired function and neurobiology.

*4. Evidence from functional MRI studies*

A few studies have examined neurophysiological differences between P-C and P-NC using functional MRI. In general, these studies have been modest in size, have studied varying paradigms and have been limited by a failure to include a cannabis-using control group and therefore require further replication. Nonetheless, they indicate differences between cannabis-using patients and those who do not use cannabis. These include differences in regional brain activation in emotional memory (prefrontal activation increased in P-C) (Bourque et al., 2013); functional connectivity of default mode and effort mode networks (Løberg et al., 2012), although an alternate resting state study showed no difference between groups (Peeters et al., 2015); differences in parietal activation in visuospatial tasks (Potvin et al., 2013); as well as differences in tasks used to interrogate addiction paradigms including attentional bias (greater amygdala activation in P-C) (Machielsen et al., 2014) and cannabis cue reactivity (greater amygdyla and thalamic activation in P-C) (Machielsen et al., 2018).

### III. Unravelling neurochemical differences between groups

*1. Evidence from the endocannabinoid system*

The interaction of exogenous phytocannabinoids is through perturbation of the endocannabinoid system (the lipid signalling system that includes cannabinoid receptors and their naturally occurring ligands). The endocannabinoid receptor system comprises the CB1 and CB2 receptors, and G-protein coupled receptors with relative, but not complete segregation of CB1 receptors to the central nervous system, whereas CB2 receptor is expressed in the peripheral nervous system and believed to be involved in inflammatory processes (Lu and MacKie, 2016). These are not the only receptors involved in the endocannabinoid system, with evidence for implication of the TRPV1 receptor in peripheral sensory neurons, peroxisome proliferator-activated receptor gamma (PPAR-γ) in peripheral tissues and other G-protein coupled receptors such as GPR18 and GPR55(Lu and MacKie, 2016; Pertwee et al., 2010). Endogenous agonists that engage cannabinoid receptors are anandamide and 2-arachidonoyl glycerol (2AG); they are synthesized from lipid precursors (Lu and MacKie, 2016), hence cannot be stored in vesicles and are synthesized ‘on demand’ (Alger and Kim, 2011; Mechoulam and Parker, 2013). For the most part, endogenous cannabinoids exert influence on pre-synaptic CB1 receptors to inhibit further neurotransmitter release (such as glutamate or GABA) via retrograde transmission, although there is also more recent evidence for non-retrograde and autocrine effects (Castillo et al., 2012). Degradation of anandamide and 2-AG occurs by the enzymes fatty acid amide hydrolyse (FAAH) and monoacylglycerol lipase (MAGL), respectively, and inhibition of degradation is related to prolonged endocannabinoid action (Mechoulam and Parker, 2013). Evidence of alteration in components of the endocannabinoid system unrelated to cannabis use in patients with psychosis (summarized by Appiah-Kusi et al., 2016) suggests that modulation of the endocannabinoid system may be implicated in psychosis risk.

Most work on understanding the phytocannabinoid–psychosis interaction has focused on the CB1 receptor, due to its widespread distribution in the brain and implication in psychotomimetic mechanisms and risk. The CB1 receptor is believed to be the most widely G-protein coupled receptor in the brain (Szabo, 2014), with CB1 receptors distributed widely distributed throughout the brain in neocortical (cingulate gyrus, frontal cortex, secondary somatosensory), hippocampal, amygdalar, cerebellar and basal ganglia areas (Mackie, 2005; Pertwee, 2008; Szabo and Schlicker, 2005). Delta-9-tetrahydrocannabinol (THC), the psychotomimetic constituent of cannabis, acts as a partial CB1 receptor agonist, with administration of THC leading to altered activation in a wide range of frontal (anterior cingulate, right inferior frontal gyri), parietal and limbic areas (midbrain, mediotemporal and ventro-striatal) (Bhattacharyya et al., 2009, 2012a, b, c; 2015a, b; Borgwardt et al., 2008; Winton-Brown et al., 2011). Increased potency of CB1 receptor agonism, either through increased THC potency or through full agonism of the CB1 receptor with synthetic cannabinoid receptor agonists has been noted to be associated with increased risk of developing psychosis and a more protracted course (Di Forti et al., 2014; Nia et al., 2016; Winstock et al., 2015). In contrast, CBD has been demonstrated to oppose the actions of THC (Bhattacharyya et al., 2010, 2012a, 2015b), is less well understood and is believed to act through a wide variety of actions including the antagonism of THC effects on the cannabinoid type 1 receptor, CB1 receptor inverse agonism, FAAH inhibition and 5HT1A activation (Parolaro et al., 2014; Pertwee, 2008). Particular interest has centred on cannabidiol as a potential treatment for psychosis, with one double-blind randomized controlled study showing non-inferiority to amisulpride after 28 days (*n*=39, cannabis users excluded), associated with concomitant increase in anandamide levels (Leweke et al., 2012), and another (*n*=88, baseline cannabis users=3/88) showing improved response versus placebo after 6 weeks as adjunctive therapy to patients’ usual medication for psychosis (McGuire et al., 2018).

Differences between patients with psychotic disorders who use cannabis and those who do not are shown in Table 3(a). Some evidence for alterations in endocannabinoid components has been demonstrated between groups. One study reported on anandamide cerebrospinal fluid (CSF) levels, finding low-frequency using patients (lifetime use less than five times) to have over 10-fold higher anandamide CSF levels compared with healthy control groups and non-using patients. The finding was not replicated in serum samples. The authors suggested that anandamide may act as a compensatory mechanism in acute psychosis, while frequent cannabis use could lead to a down-regulation of anandamide signalling to explain the results (Leweke et al., 2007). Another, albeit small, study (*n*=12) showed alterations in lipid precursors of endocannabinoids between users and non-users; however, given the small size this needs further replication (Monterrubio et al., 2006).

Most work in this area has been undertaken on the CB1 receptor. One early study showed increased CB1 receptor binding in the dorsolateral prefrontal cortex in P-C versus P-NC (Dean et al., 2001). However, since then a number of studies have not demonstrated any differences between groups (Ceccarini et al., 2013; Deng et al., 2007; Eggan et al., 2008, 2010; Ranganathan et al., 2016; Volk et al., 2014; Zavitsanou et al., 2004). These studies are mostly small in size and the two in vivo studies were not designed to test P-C versus non-PC (Ceccarini et al., 2013; Ranganathan et al., 2016); this notwithstanding, many of these studies were able to demonstrate altered CB1 receptor expression in patients compared with controls.

Finally, some work has demonstrated evidence of altered genotype by cannabis effects relating to white matter deficits. These found sensitivity for the following genotypes for the cannabinoid 1 receptor gene: rs12720071 to white matter damage and neurocognitive impairment, and for the MAPK14 gene (believed to be involved in CBR1 apoptosis induced by THC) for rs12199654. Both genotypes were demonstrated to have independent and additive effects (Ho et al., 2011; Onwuameze et al., 2013).

*2. Evidence from the dopamine system*

One would expect cannabis, a robustly implicated risk factor for psychosis, to affect dopamine signalling in the human brain. The dopamine hypothesis is the pre-eminent explanation for psychotic illness (Howes and Kapur, 2009). Indeed, in preclinical models THC has been shown to induce increases of dopamine neuron firing and dopamine levels in key mesolimbic areas including the ventral tegmental area as well as the nucleus accumbens and striatum (Cheer et al., 2004; French et al., 1997; Ginovart et al., 2012; Tanda et al., 1997). This relationship appears to be bidirectional, with preclinical evidence that dopamine modulates striatal endocannabinoid release (Giuffrida et al., 1999; Kreitzer, 2005).

Yet in human studies the evidence is far less conclusive. Studies using functional MRI in healthy volunteers have demonstrated that transient psychotic symptoms induced by experimental administration of THC is associated with its effects on activation in key brain areas rich in dopaminergic innervation, such as the striatum and midbrain (Bhattacharyya et al., 2009, 2012a, b). However, a systematic review of data from 25 human studies which directly investigated various aspects of the dopamine signalling pathway in over 568 participants, of which 244 belonged to the cannabis or cannabinoid exposure group (Sami et al., 2015), suggests a tenuous link between cannabinoid exposure and alterations in the dopaminergic signalling system. Even allowing for less precise estimations of dopamine in man, as compared with in animals, collectively current evidence suggests that while dopamine signalling alterations may be a consequence of cannabis use, the link may not be direct. As previously noted, there are limitations to extrapolating data from healthy controls to patients. Acute challenge studies of THC in healthy volunteers have revealed mixed results with no difference between groups (Barkus et al., 2011; Stokes et al., 2012) and striatal release noted in one study (Bossong et al., 2009) or increased dopamine transmission in post-hoc analysis (Bossong et al., 2015; Stokes et al., 2010). Most studies have not studied patients with psychotic disorder, but when patients with cannabis use have been included there appears to be evidence for blunting of dopaminergic response rather than sensitization, as may have been expected (Bloomfield et al., 2014; van de Giessen et al., 2016; Wiers et al., 2016), although a stress task found no difference between cannabis users and controls (Mizrahi et al., 2013).

Relatively few studies have looked specifically at patients with psychosis who use cannabis versus those who do not use. One study undertaking post-mortem autoradiography found no differences in dopamine active transport affinity (a marker of dopaminergic neurons) or tyrosine hydroxylase between P-C and P-NC; however, this was a small study and classified participants only on blood THC levels at time of death (Dean et al., 2003). Another study looking at peripheral markers at admission found dopamine metabolites in the P-C group compared with other groups, but has not been replicated and was compromised by small sample sizes (P-C, *n*=5) (Bowers and Kantrowitz, 2007). Three studies have undertaken scintillography in clinical samples. Using SPECT one study found no differences in D2 receptor binding in first-episode patients who used cannabis and those who did not (Safont et al., 2011). Interestingly, consistent with blunting shown in cannabis users (discussed above), in a clinical high-risk group administered a stress task, heavy cannabis users (use of at least three times per week) were shown to have blunted dopamine release compared with non-users (Mizrahi et al., 2014). One study has suggested that there may be differential responses based upon genetic risk of psychotic disorder (Kuepper et al., 2013). Kuepper and colleagues administered THC or placebo to eight patients, seven relatives and nine healthy controls, finding significant striatal displacement of fallypride (indicating synaptic dopamine release) in patients and relatives but not controls. This may indicate that patients or those at genetic risk have an increased sensitivity to the effects of cannabis; further studies are required to replicate this and delineate the extent of this phenomenon.

*3. Evidence from the glutamatergic and GABAergic systems*

If dopamine abnormalities do not fully explain psychosis linked to cannabis use, an alternate explanation must be sought. CB1 receptor activation, the mechanism through which THC induces psychotic symptoms under experimental conditions, mediates inhibition of glutamate and γ-aminobutyric acid (GABA) through retrograde transmission in cortical, hippocampal and midbrain regions (Mechoulam et al., 2014). While these may therefore seem valid alternative pathways, little work has been undertaken to date to determine whether glutamatergic/GABAergic abnormalities are associated with cannabis use in psychosis. Several lines of evidence indicate glutamatergic dysfunction in psychotic illness independent of cannabis exposure. These include: (1) drug-induced models with NMDA receptor antagonists ketamine and PCP producing schizophrenia-like psychosis; and (2) Magnetic resonance spectroscopy (MRS) studies indicating altered glutamatergic metabolites in psychosis patients compared with controls (Merritt et al., 2016).

To date there appears some suggestion that cannabis use, independent of psychosis, may be associated with perturbation of the glutamatergic system. Decreased glutamatergic indices in cortical and subcortical areas in cannabis users compared with non-users have been reported (Colizzi et al., 2016). Furthermore, preclinical work has implicated cannabinoid receptor activation in the reduction of long-term potentiation of glutamatergic transmission through NMDA receptor hypofunction at the hippocampus, which is associated with deficits in learning and memory (Sánchez-Blázquez et al., 2013). We are only aware of one study which has reported on glutamate levels in patients who use cannabis and those who do not (Rigucci et al., 2017). Rigucci and colleagues found lower glutamatergic indices in the prefrontal cortex in cannabis-using versus non-using patients. Interestingly, in their sample the cannabis users were more rather than less cognitively impaired as compared with non-cannabis-using patients. Further studies are required to determine whether these results are replicable in other cohorts. MMN has also been used to interrogate glutamatergic perturbation, as it is well demonstrated that NMDA antagonists such as ketamine induce alterations in MMN (Avissar and Javitt, 2017). As noted, two studies have indicated altered MMN in P-C, indicating possible underlying glutamatergic dysfunction (Pesa et al., 2012; Rentzsch et al., 2011). Glutamate may thus be a promising avenue for further investigation.

Evidence of GABAergic deficits mimicking psychotic-like symptoms and deficits in GABAergic markers in patients on post-mortem studies (Lewis et al., 2005) supports a GABAergic theory of schizophrenia. However, a meta-analysis of MRS GABA imaging has revealed no difference between schizophrenia patients and controls (Schür et al., 2016). Interestingly, a recent challenge study did demonstrate enhanced psychotomimetic effects of synthetic THC when co-administered with iomazenil, a GABA antagonist (Radhakrishnan et al., 2015). This may suggest a role for the GABAergic system in inhibiting psychotic-like symptoms induced by THC. Nevertheless, there appears to be less support for a GABAergic in contrast to a glutamatergic alteration as a potential mechanism linking cannabis and psychosis. To date no studies have directly reported on GABAergic differences between P-C and P-NC. However, GABA function has been indexed by studies on cortical inhibition which is believed to be GABA-A-mediated (Stagg et al., 2011). The two studies undertaken to date have given opposing results (Goodman et al., 2017; Wobrock et al., 2010) which may be due to differences in the definition of the cannabis-using group (dependence or use ≥20 times) or other methodological differences, and further work is required to determine the effect of cannabis use on the GABAergic system in psychosis.

#### Future approaches

Collectively, there is therefore a compelling case for investigating glutamatergic indices in psychosis patients with cannabis use. Interaction with the GABAergic system also requires further elaboration. Future work should therefore investigate whether cannabis use is associated with alterations in glutamatergic and/or GABAergic indices. These can be investigated through MRS and single photon emission tomography (SPET) or positron emission tomography (PET) approaches. MRS is widely used in investigating both glutamate and GABA levels in psychosis, but is limited by its inability to differentiate between metabolic and neurotransmitter pools of glutamatergic markers (Poels et al., 2013). PET/SPET have been limited by lack of available tracers; however, radioligands for both GABA and glutamate systems offer advantage of specificity and are amenable to dynamic challenge studies (Finnema et al., 2015). Advances in MRS image acquisition and radiotracer development hold promise for future research in this area (Finnema et al., 2015; Poels et al., 2013).

Regarding the endocannabinoid system, particular promise may be related to the effects of cannabidiol. As yet no randomized controlled trial has determined whether there is an increased efficacy and acceptability of CBD as a medication for psychosis in cannabis users and the neurobiological mechanisms, including perturbation of the endocannabinoid system which may underpin this.

Imaging techniques may be applied to differentiate cannabis-using and non-using patients across the trajectory of illness, including clinical high-risk groups, unmedicated and medicated patients with early psychosis and patients with enduring illness. Longitudinal study design will likely help establish the precise relationship between cannabis use, alterations in the glutamate/GABA signal and outcome. Understanding the neurochemical mechanisms would enable alternative treatment strategies to be developed for this patient group, or may indeed point towards a ‘endocannabinoid dysfunction pathway’ to psychosis pathway, not fully explained by dopaminergic, glutamatergic or GABAergic changes.
